# Origin of Lactose Fermentation in *Kluyveromyces lactis* by Interspecies Transfer of a Neo-functionalized Gene Cluster during Domestication

**DOI:** 10.1016/j.cub.2019.10.044

**Published:** 2019-12-16

**Authors:** Javier A. Varela, Martina Puricelli, Raúl A. Ortiz-Merino, Romina Giacomobono, Stephanie Braun-Galleani, Kenneth H. Wolfe, John P. Morrissey

**Affiliations:** 1 School of Microbiology, Centre for Synthetic Biology and Biotechnology, Environmental Research Institute, APC Microbiome Ireland, University College Cork, Cork T12YN60, Ireland; 2 Department of Biotechnology, Delft University of Technology, Van der Maasweg 9, 2629 Delft, the Netherlands; 3 UCD Conway Institute, School of Medicine, University College Dublin, Dublin 4 D04 C7X2, Ireland

**Keywords:** introgression, domestication, lactose, fermentation, yeast, neofunctionalization, cluster, lactis, marxianus, *Kluyveromyces*

## Abstract

Humans have used yeasts to make cheese and kefir for millennia, but the ability to ferment the milk sugar lactose is found in only a few yeast species, of which the foremost is *Kluyveromyces lactis* [1]. Two genes, *LAC12* (lactose permease) and *LAC4* (lactase), are sufficient for lactose uptake and hydrolysis to glucose and galactose [2]. Here, we show that these genes have a complex evolutionary history in the genus *Kluyveromyces* that is likely the result of human activity during domestication. We show that the ancestral Lac12 was bifunctional, able to import both lactose and cellobiose into the cell. These disaccharides were then hydrolyzed by Lac4 in the case of lactose or Cel2 in the case of cellobiose. A second cellobiose transporter, Cel1, was also present ancestrally. In the *K. lactis* lineage, the ancestral *LAC12* and *LAC4* were lost and a separate upheaval in the sister species *K. marxianus* resulted in loss of *CEL1* and quadruplication of *LAC12*. One of these *LAC12* genes became neofunctionalized to encode an efficient lactose transporter capable of supporting fermentation, specifically in dairy strains of *K. marxianus*, where it formed a *LAC4-LAC12-CEL2* gene cluster, although another remained a cellobiose transporter. Then, the ability to ferment lactose was acquired very recently by *K. lactis* var. *lactis* by introgression of *LAC12* and *LAC4* on a 15-kb subtelomeric region from a dairy strain of *K. marxianus*. The genomic history of the *LAC* genes shows that strong selective pressures were imposed on yeasts by early dairy farmers.

## Highlights

• Early farmers domesticated the milk yeast *Kluyveromyces lactis*
• A dairy lineage of *Kluyveromyces marxianus* donated the *LAC* genes to *K. lactis*
• A new *Kluyveromyces*-specific gene cluster for utilizing cellobiose was identified
• *K. marxianus* carries an integrated cellobiose and lactose utilization gene cluster

## Graphical Abstract

**Figure:**
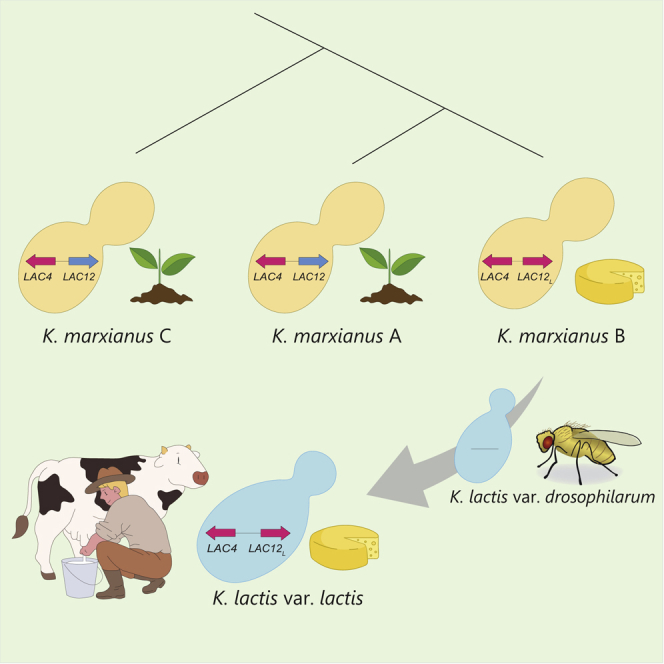

## Teaser

It is well known that humans domesticated brewer’s yeast, and now, Varela et al. report that another yeast is also the product of human activity. They show that an insect-associated, lactose-negative progenitor of the milk yeast *Kluyveromyces lactis* acquired the genes that enable lactose fermentation from a dairy-adapted population of *K. marxianus*.

## Results and Discussion

### Genomes, Phylogeny, and Phenotypes of *Kluyveromyces* Species

The yeast genera *Kluyveromyces* and *Saccharomyces*, which diverged about 150 mya, both contain species that are important producers of fermented foods or beverages or that serve as hosts for production of metabolites and proteins for biotechnology [2, 3]. The capacity to grow on lactose as a sole carbon source is a defining trait in the food yeasts *Kluyveromyces lactis* and *Kluyveromyces marxianus*.

The lactose utilization system was elucidated in *K. lactis* and depends primarily on two neighboring genes, *LAC12* and *LAC4* [2, 4, 5]. Lac12 is a membrane permease that imports lactose into the cell, and Lac4 is an intracellular lactase (β-galactosidase) that hydrolyzes lactose into the easily catabolized monosaccharides glucose and galactose. The kinetics of uptake of lactose by Lac12 and its hydrolysis by Lac4 are sufficient to allow fermentative growth of *K. lactis* [6] and dairy isolates of *K. marxianus* [7] in oxygen-limiting conditions. *K. lactis* and *K. marxianus* are often associated with fermented dairy products, such as artisan cheese and kefir. *K. marxianus* can also be isolated from plants and other habitats [7, 8]. Two varieties of *K. lactis* are recognized: *K. lactis* var. *lactis*, which is milk associated, and *K. lactis* var. *drosophilarum*, which is insect associated [1]. Our previous population studies of *K. marxianus* found three distinct genomic haplotypes (A, B, and C). Haplotype B is dairy associated and carries a *LAC12* allele that encodes a protein variant, Lac12_L_, with enhanced capacity to transport lactose [7, 9]. There are four *LAC12* genes in the *K. marxianus* genome, but only the Lac12_L_ variant has efficient lactose-uptake properties [9].

The six currently recognized species in the genus *Kluyveromyces* vary widely in their ability to metabolize lactose [1]. Three phenotypic groups can be described. First, only *K. lactis* var. *lactis* and dairy strains of *K. marxianus* (B haplotype) can ferment lactose. Second, *K. dobzhanskii* and *K. lactis* var. *drosophilarum* are lactose negative, unable to utilize this sugar at all. A third phenotypic group is formed by *K. aestuarii*, *K. nonfermentans*, and *K. wickerhamii* and non-dairy isolates of *K. marxianus* (A and C haplotypes), which are Kluyver effect positive for lactose—meaning that they can respire, but not ferment the sugar [10]. Because these three phenotypic groups do not correspond to phylogenetic clades, and because the trait of biotechnological interest (lactose fermentation) is polymorphic for presence/absence in both *K. lactis* and *K. marxianus*, we were motivated to investigate the origin and evolution of the *LAC* genes.

**Figure 1:**
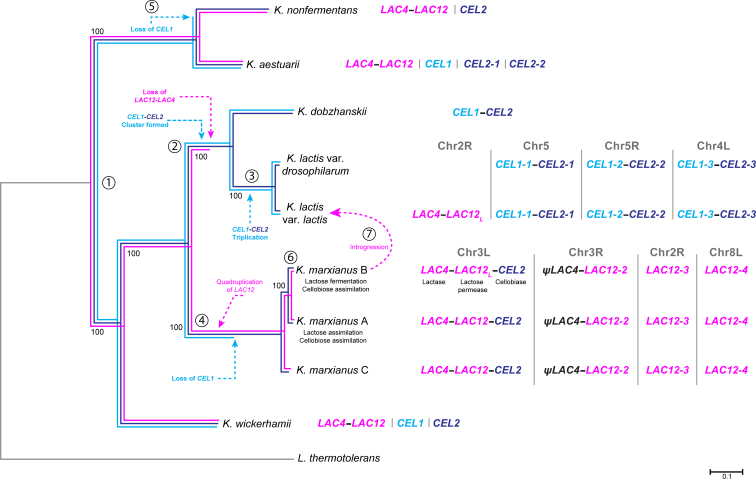
Summary of Evolutionary Steps in Lactose and Cellobiose Utilization in the Genus *Kluyveromyces* Colored branches on the phylogenetic tree trace the history of the *LAC4-LAC12* gene cluster (magenta), *CEL1* (cyan), and *CEL2* (dark blue). Dashed arrows mark key evolutionary steps, including gene duplications, losses, and relocations. Numbers refer to specific events that are discussed in the main text. The dashed magenta arrow shows the introgression of the *LAC12*_*L*_ gene and the neighboring *LAC4* from a *K. marxianus* haplotype B strain into a lactose negative progenitor of *K. lactis* var. *lactis*, leading to the modern species that is now able to assimilate and ferment lactose. The *LAC* and *CEL* genotype is shown for every species. The chromosome numbers for the *K. lactis* species refers to chromosomes in *K. lactis* var. *lactis* CBS2359. Dashes between gene names indicate genes that are clustered in the genome. See also Figure S1 and Table S1.

Genome sequences are available for all six *Kluyveromyces* species, including *K. marxianus* haplotypes A, B, and C and the type strain of *K. lactis* var. *lactis* (CBS2359). We sequenced the type strain of *K. lactis* var. *drosophilarum* (CBS2105) and assembled it into six complete chromosome sequences. Comparison to CBS2359 reveals genome-wide nucleotide sequence identity of 95.4% and karyotypes that differ by two inversions and four reciprocal translocations. A phylogenomic tree of the six species (Figure S1) gives a topology that agrees with a recent phylogenomic study [11]. The two varieties of *K. lactis* are seen to be very closely related, as are the three haplotypes of *K. marxianus* (Figure 1).

### The *LAC* Genes of *K. lactis* var. *lactis* Were Acquired by Introgression from *K. marxianus*

**Figure 2:**
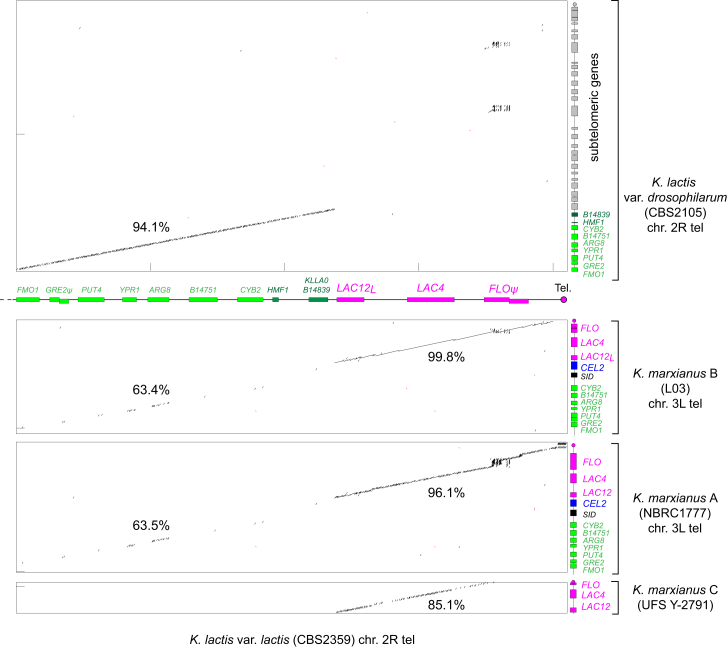
The *LAC4-LAC12* Gene Region in *K. lactis* var. *lactis* Was Formed by Introgression of a 15-kb Subtelomeric Region from *K. marxianus* Four dot matrix plots are shown. The x axis in all the plots is a 35-kb region beside the telomere of chromosome 2R of *K. lactis* var. *lactis*, including the genes *LAC12*, *LAC4*, and a flocculin pseudogene (*FLOψ*). This region is compared to *K. lactis* var. *drosophilarum* in the uppermost plot and to *K. marxianus* (haplotypes A, B, and C) in the three lower plots. The introgression into *K. lactis* var. *lactis* replaced a subtelomeric region containing approximately 26 genes (gray) with one containing 3 genes (magenta). Numbers show percent DNA sequence identity in CLUSTALΩ alignments of the regions from *FMO1* to *CYB2* and from *LAC12* to the telomere between genomes. The genes *HMF1* and *KLLA0B14839* are present only in *K. lactis* in this region, and the genes *CEL2* and *SID* (a putative siderophore transporter) are present only in *K. marxianus.* The plots were made using DNAMAN (https://www.lynnon.com) with a criterion of 17 matches per 20-bp window. See also Table S1.

The genome sequence of *K. lactis* var. *drosophilarum* does not contain any *LAC12* or *LAC4* genes, consistent with a previous report based on Southern blotting [12] and with the inability of this strain to grow on lactose. The *LAC12-LAC4* gene cluster is located in a subtelomeric region in both *K. lactis* and *K. marxianus*, so we used dot matrix plots to compare this region between *K. lactis* var. *lactis* and either *K. lactis* var. *drosophilarum* or *K. marxianus* haplotypes A, B, and C (Figure 2). The genomic region between *FMO1* and *CYB2* (green in Figure 2) is orthologous between *K. lactis* and *K. marxianus*, even though its DNA sequence identity is low (63%). Between the two *K. lactis* varieties, the high DNA sequence identity (94.1%), which extends over most of the chromosome, terminates abruptly after the gene (*KLLA0B14839*) located immediately before the *LAC12-LAC4* gene cluster and the telomere (Figure 2, upper panel). Instead, the region from *LAC12* to the telomere in *K. lactis* var. *lactis* has very high similarity to the corresponding region of *K. marxianus* (Figure 2, lower panels). Moreover, it has higher sequence identity to the B haplotype of *K. marxianus* (99.8% identity; only 31-nt differences in 13,961 bp aligned) than to the A and C haplotypes (96.1% and 85.1% identity, respectively). Phylogenetic analysis of the proteins encoded by *LAC12* and *LAC4* confirms that these genes in *K. lactis* var. *lactis* are more closely related to their homologs in a *K. marxianus* B-haplotype strain than to A- or C-haplotype strains (Figures S2 and S3).

These results show that the telomere-proximal region, including the *LAC* genes, was transferred between the two *Kluyveromyces* species, confirming a hypothesis by Naumov that *K. lactis* might have obtained its *LAC* genes by horizontal gene transfer [13]. The donor was a B-haplotype (dairy) strain of *K. marxianus*, and the recipient was a *K. lactis* strain that became the progenitor of *K. lactis* var. *lactis*. The transferred region (magenta in Figure 2) was approximately 15 kb and included *LAC12*_*L*_, *LAC4*, and a flocculin (*FLO*) gene that has since acquired frameshift mutations and is thus a pseudogene. The telomere was also transferred during the introgression event. We infer that the transfer replaced a previous subtelomeric region in *K. lactis* that resembled the current subtelomere of *K. lactis* var. *drosophilarum*, which consists of a 70-kb region containing 26 genes (gray in Figure 2). The most likely mechanism of DNA transfer was introgression, i.e., interspecies mating between *K. marxianus* and *K. lactis*, followed by repeated backcrossing to *K. lactis*, although the rearrangement was more complex than a meiotic crossover in the last orthologous gene, *CYB2* (Figure 2).

### The *CEL1-CEL2* Gene Cluster Encodes a Newly Identified Cellobiose Utilization System in *Kluyveromyces*

The gene immediately beside the *LAC12*-*LAC4* gene cluster in *K. marxianus* has features suggesting that it codes for a β-glucosidase. There are no other candidate β-glucosidase genes in the *K. marxianus* genome, so we hypothesized that this gene, which we named *CEL2*, encodes a cellobiase that enables *K. marxianus* to grow on the disaccharide cellobiose by hydrolyzing it to glucose. It has previously been shown that *K. lactis* Lac12 (Lac12_L_) is able to transport cellobiose when expressed in *S. cerevisiae* [14, 15], so we wondered whether the juxtaposition of *LAC12* and *CEL2* in the *K. marxianus* genome, coding respectively for proteins that can potentially import and hydrolyze cellobiose, constitutes a functional gene cluster. But if this is the case, what is the system for utilizing cellobiose in species such as *K. lactis* var. *drosophilarum* and *K. dobzhanskii* that can grow on this sugar but do not carry *LAC12*?

**Figure 3:**
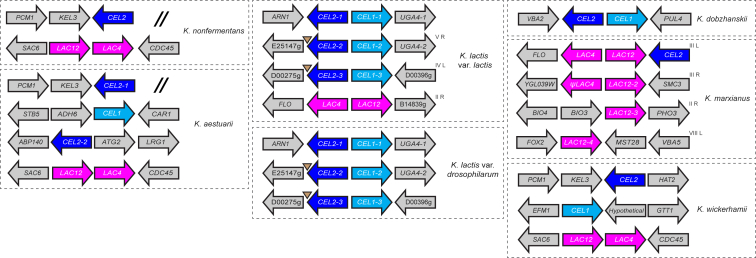
Organization of *LAC* and *CEL* Genes in the Genomes of *Kluyveromyces* Species and Varieties Dashed lines delimit the genes that belong to each species. The *LAC* genes are shown in magenta. *CEL1* and *CEL2* are shown in cyan and dark blue, respectively. Missing information due to incomplete genome assembly is indicated with a double slash symbol in the *K. nonfermentans* and *K. aestuarii* panels. LTR elements are represented by inverted triangles. The organization of the *K. marxianus* genes is based on the CBS397 assembly [7]. *ψLAC4* is a conserved pseudogene and *LAC12-4* is a pseudogene only in some strains, and variability both in the sequence of Lac4 and in the genomic organization in this telomere of chromosome 8 is seen between strains [9]. Superscript text indicates the position (chromosome number and arm) of the *LAC* and *CEL* genes in *K. marxianus* and the *K. lactis* var. *lactis* variants. Loci in *K. lactis* var *lactis* and *K. lactis* var. *drosophilarum* are syntenic, but reciprocal translocations may have changed chromosome numbers. See also Figures S2 and S3 and Table S1.

*CEL2* genes are present in all six *Kluyveromyces* species, with some having multiple paralogs (Figure 3). Phylogenetic analysis shows that the putative cellobiase Cel2 groups with fungal β-glucosidases, whereas the lactase Lac4 forms a clade with β-galactosidases from other fungi and bacteria (Figure S2). In the *K. dobzhanskii/K. lactis* clade, we also found a putative sugar transporter gene that we named *CEL1*. This gene is adjacent to *CEL2*, which is suggestive of another functional gene cluster. *K. wickerhamii* and *K. aestuarii* also have *CEL1* genes, but they are not linked to *CEL2.* In contrast, *CEL1* is absent in *K. marxianus* and *K. nonfermentans*. Comparison to known cellobiose and lactose transporters from other fungi shows that Cel1 groups with cellobiose transporters, whereas Lac12 groups with fungal lactose permeases (Figure S3). Based on these phylogenetic analyses and the previous report that Lac12 can transport cellobiose as well as lactose [14, 15], we hypothesized that (1) Cel1 and Cel2 together constitute a system for import and hydrolysis of cellobiose and (2), in some species, Lac12 serves as cellobiose transporter that is an alternative to Cel1.

**Figure 4:**
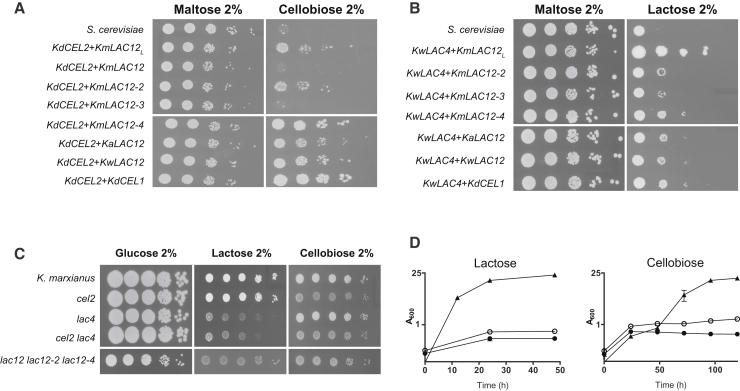
Functional Analyses Confirm that the *LAC* and *CEL* Genes Encode Functional Sugar Assimilation Systems The function of the putative permeases and hydrolases was assessed by heterologous expression in *S. cerevisiae* and CRISPR-Cas9-induced mutagenesis in *K. marxianus*. (A and B) *S. cerevisiae* strains transformed with different combinations of putative hydrolyses (*LAC4* or *CEL2*) and permeases (*LAC12* alleles or *CEL1*) were assessed for growth on SC medium with cellobiose (A) or lactose (B) as the carbon source. The control strain is *S. cerevisiae* transformed with the empty pGREG505 and p426 plasmids and growth on maltose assessed as a positive control. The function of the *LAC* and *CEL* genes was also evaluated in *K. marxianus*. Targeted mutants of the *LAC12* genes, *LAC4* and *CEL2*, were constructed using the CRISPR-Cas9 system in *K. marxianus* NBRC 1777. (C and D) These mutants were assessed for growth on plates (C) and in liquid (D) in mineral medium with the sole carbon sources indicated. Curves are the mean of three replicates. Legend: close triangles, wild-type; closed circle, *cel2 lac4*; open circle, *lac12 lac12-2 lac12-4.* See also Figure S4 and Tables S1–S4.

To test these hypotheses, we cloned candidate transporter genes (*LAC12* or *CEL1*) and expressed them in a *S. cerevisiae* strain co-expressing *CEL2* from *K. dobzhanskii* (Figure 4A). Co-expression of *K. dobzhanskii CEL1* and *CEL2* conferred growth on cellobiose, proving that these genes encode a functional cellobiose utilization system: Cel1 can transport cellobiose and Cel2 is a cellobiase. Co-expression of *CEL2* with the single *LAC12* gene from either *K. aestuarii* or *K. wickerhamii* also enabled *S. cerevisiae* to grow on cellobiose, demonstrating that these Lac12 proteins can transport cellobiose. This is also the case for *K. marxianus LAC12*_*L*_, *LAC12-2*, and *LAC12-4*, but neither *LAC12-3* nor *LAC12* from an A-haplotype strain are efficient cellobiose transporters (Figure 4A).

To assess lactose transport, *K. wickerhamii LAC4* was co-expressed in *S. cerevisiae* with putative transporters (Figure 3B). The lactase activity of *K. wickerhamii* Lac4 was confirmed by co-expression with *K. marxianus LAC12*_*L*_, but co-expression with other *LAC12* genes or with *CEL1* led to little growth. This result agrees with previous data that, in *K. marxianus*, only the B-haplotype allele *LAC12*_*L*_ encodes a functional lactose transporter [9] and suggests that the protein encoded by *LAC12* in other *Kluyveromyces* species is a poor lactose transporter. This observation is also consistent with data that *K. aestuarii* and *K. wickerhamii* grow slowly on lactose by respiration and cannot ferment it (Kluyver effect positive) [16, 17]. The data also demonstrate that Cel1 is specific for cellobiose and is unable to transport lactose.

### Cellobiose and Lactose Uptake Is Mediated by Lac12 Transporters in *K. marxianus*

Our data indicate that there are two ancestral disaccharide utilization systems in the genus *Kluyveromyces*: Cel1 and Cel2, which import and hydrolyze cellobiose, and Lac12 and Lac4, which import and hydrolyze lactose. Lac12, however, is bifunctional, able to transport both lactose and cellobiose in heterologous expression assays (Figure 4A). Because the Cel1 transporter has been lost in *K. marxianus*, we hypothesized that cellobiose transport in this species is carried out exclusively by Lac12. To investigate whether *K. marxianus* Lac12 has this proposed bifunctionality and to confirm the separate enzymatic functions of Lac4 and Cel2, *K. marxianus* mutants were constructed using CRISPR-Cas9-induced nonsense mutation and analyzed for growth on lactose or cellobiose (Figure 4C). Comparing growth first on solid medium, the *cel2* mutant grows on lactose, but not cellobiose; the *lac4* mutant grows on cellobiose, but not lactose; and the *cel2 lac4* mutant grows on neither lactose nor cellobiose (Figure 4C). The faint apparent growth in these plate assays is background due to dead cells, a point confirmed by the failure of the *cel2 lac4* double mutant to grow on either disaccharide in liquid medium (Figure 4D). Because heterologous expression (Figure 4A) indicated that any of the Lac12_L_, Lac12-2, or Lac12-4 proteins was able to transport cellobiose, a *K. marxianus* triple mutant lacking all three genes was assessed (Figures 4C and 4D). The triple mutant was unable to grow on either lactose or cellobiose, confirming the dual function of the *K. marxianus LAC12* gene family and establishing that there is no other unidentified cellobiose transporter in *K. marxianus*. Thus, in *K. marxianus*, Lac12 is a bifunctional transporter that can transport either lactose or cellobiose, which is then cleaved into monosaccharides by the dedicated hydrolytic enzyme, Lac4 or Cel2 (Figure S4).

### Reconstruction of the Evolutionary Trajectory of the *LAC* and *CEL* Genes in *Kluyveromyces* Species

Our analyses enable us to reconstruct the evolutionary history of the genes for lactose and cellobiose utilization in the *Kluyveromyces* genus (Figure 1). Because *K. wickerhamii* and *K. aestuarii* are located on branches that diverged at the base of the genus, it is likely that the similar genomic organization of the *CEL* and *LAC* genes in these species (Figure 3) represents the ancestral state in the genus. Thus, *LAC12* and *LAC4* were already contiguous in the genome of the ancestor (beside *SAC6*; Figure 3), but *CEL1* and *CEL2* were not contiguous. This ancestor is labeled as point 1 in Figure 1. On the branch that led to the *K. dobzhanskii/K. lactis* clade, there were several reorganizations of the *CEL* genes (point 2). One of these brought *CEL1* and *CEL2* together to assemble a gene cluster for cellobiose utilization. This *CEL* gene cluster became located in a subtelomere in *K. lactis* and was later triplicated during a large-scale amplification of *K. lactis* subtelomeres (point 3) [18]. In contrast, *CEL1* was lost from the *K. marxianus* branch (point 4) and also from *K. nonfermentans* (point 5). Separate reorganization of the *LAC* genes took place. The *LAC* cluster was lost from the *K. dobzhanskii/K. lactis* branch (point 2). In the *K. marxianus* branch (point 4), it was rearranged such that *LAC12* and *LAC4* are now divergently transcribed from a common promoter (Figure 3). The *LAC* gene cluster became quadruplicated onto multiple *K. marxianus* subtelomeres, but *LAC4* was lost or degenerated into a pseudogene in all the copies except the one at the subtelomere of chromosome 3L. Also at point 4, *CEL2* was relocated to the same subtelomere, forming the three-gene cluster *LAC4-LAC12-CEL2*. Within the species *K. marxianus*, there was divergence into distinct haplotypes and the *LAC12* allele in haplotype B (*LAC12*_*L*_) acquired amino acid changes that improved its ability to transport lactose (point 6). Then, recently, a 15-kb subtelomeric region containing the *LAC* genes introgressed from a *K. marxianus* B-haplotype strain into *K. lactis* (point 7), splitting the latter species into two varieties and restoring lactose utilization (now fermentable because of efficient uptake) to *K. lactis* var. *lactis*. The transferred region contained *LAC12*_*L*_ and *LAC4* from the three-gene cluster, but not *K. marxianus CEL2* (*K. lactis* already had native *CEL2* genes in its *CEL1-CEL2* clusters; Figure 3).

From the genomic data, it is possible to say that the host for the introgression was a *K. lactis* strain resembling the insect-associated species *K. lactis* var. *drosophilarum*. We expect that further analysis of genetic diversity in *K. lactis* will show that strains containing the introgressed *LAC* genes form a subclade that lies within a more diverse set of insect-associated *K. lactis* strains that cannot grow on lactose. The introgression can be inferred to be a recent event on evolutionary timescales because of the low number of nucleotide differences between the donor and recipient. Using Rolland and Dujon’s method for yeast molecular clocks [19], we estimate that the introgressed DNA diverged from the *K. marxianus* B-haplotype approximately 3.7 million generations ago, which corresponds to between 3,700 and 37,000 years ago, depending on the number of yeast generations assumed per year. This is close to or within the timescale for the emergence of agriculture. The milk-producing animals cow, sheep, and goat were all domesticated between 8,000 and 10,000 years ago [20], and paleoproteomic analysis of dental calculus has shown that humans were consuming milk, most likely as cheese or other fermented products, by 5,500 years ago [21]. Because our estimate for the age of the *LAC* gene introgression extends into the period of milk animal domestication, it is plausible that selection for the introgression was the result of human activity during production of a fermented milk product, such as cheese or kefir. There are some parallels between this event and the recently reported trans-species introgression of *GAL* genes from an unknown donor *Saccharomyces* species into milk- or cheese-associated strains of *S. cerevisiae*, which utilize the galactose and glucose formed by bacterial hydrolysis of lactose [22, 23]. Together, the introgressions in both *Kluyveromyces* and *Saccharomyces* point to strong selective pressures imposed on yeasts by early farmers for the ability to ferment animal milk.

## STAR★Methods

### Key Resources Table

**Table:** 

REAGENT or RESOURCE	SOURCE	IDENTIFIER
**Deposited Data**		

*K. lactis* var. *drosophilarum* genome assembly	This paper	GenBank: GCA_007993695.1
*K. marxianus* L03 genome assembly	This paper	GenBank: GCA_008000265.1

**Experimental Models: Organisms/Strains**		

*Kluyveromyces* and *S. cerevisiae* strains	N/A	See Table S1
Genotype of *K. marxianus* mutants	This study	See Table S4

**Oligonucleotides**		

Oligonucleotides	Sigma	See Table S3

**Recombinant DNA**		

Plasmids	N/A	See Table S2

**Software and Algorithms**		

SPAdes v3.11.1	N/A	http://cab.spbu.ru/files/release3.11.1/
RAxML-NG v0.8.1	N/A	https://github.com/amkozlov/raxml-ng/releases
MUMmer	N/A	http://mummer.sourceforge.net/

### Lead Contact and Materials Availability

Further information and requests for resources and reagents should be directed to and will be fulfilled by the Lead Contact, John P. Morrissey (j.morrissey@ucc.ie).

### Experimental Model and Subject Details

Experiments were conducted using *S. cerevisiae* and *Kluyveromyces* yeasts. All the yeast strains used in this study are listed in Table S1. *Kluyveromyces* strains were purchased from the Westerdijk Fungal Biodiversity Institute and *S. cerevisiae* EBY.VW4000 was kindly provided by Dr Eckhard Boles, Goethe University Frankfurt, Germany. This strain is deleted for 17 hexose transporter genes but can grow on the disaccharide, maltose, which is therefore used as a control in the experiments. *S. cerevisiae* EBY.VW4000 was used for heterologous expression of *Kluyveromyces* genes. *K. marxianus* NBRC1777 (haploid, haplotype A) was used to construct mutants for transporters and hydrolases and *K. marxianus* CBS397 (diploid, haplotype AB) was used for phenotypic tests and as the source of template DNA to clone *LAC* and *CEL* genes. Genome sequences of *K. marxianus* NBRC 1777 (haploid, haplotype A), *K. marxianus* L03 (triploid, haplotype BBB), and *K. marxianus* Y-UFS 2791 (haploid, haplotype C) were used for comparison of genomic structure between *K. marxianus* and *K. lactis* spp.

### Method Details

#### Strains and culture conditions

The *Kluyveromyces* species were routinely grown in YPD broth (1% yeast extract, 2% peptone, 2% glucose). For sugar utilization experiments the strains were grown on mineral medium [24] supplemented with 2% glucose, then washed twice with sterile water, diluted to A_600_ 1, diluted serially (10-fold) and spotted onto minimal medium plates containing 2% glucose, lactose, raffinose or cellobiose (Thermo-Fisher Scientific, MA, USA) as the sole carbon source. For growth curves, the *K. marxianus* mutants were grown overnight on minimal medium and transferred to fresh medium containing the same carbon source to an A_600_ of 0.1. Growth was monitored by measuring A_600_ over time. For selecting transformants, *K. marxianus* was grown on YPD plates supplemented with 200 μg/mL^-1^. Hygromycin B (Sigma-Aldrich, MI., USA). *S. cerevisiae* EBY.VW4000 was used in heterologous expression experiments. The strain was grown on synthetic complete (SC) medium (1.7 g L^-1^ yeast nitrogen base, 5 g L^-1^ ammonium plus synthetic complete drop-out lacking uracil and L-leucine) supplemented with 2% maltose. In experiments testing the function of putative transporters, the *S. cerevisiae* strains containing different plasmids were grown on SC maltose, serially diluted and spotted on SC plates containing maltose, lactose or cellobiose to a final concentration of 2%. All the yeast strains in this study were grown at 30°C with 200 rpm agitation. *E. coli*, used for cloning experiments, was grown in LB medium (0.5% yeast extract, 1% bactopeptone, 1% NaCl) supplemented with 100 μg/mL ampicillin.

#### Heterologous expression

The *CEL* and *LAC* genes were cloned and expressed in *S. cerevisiae* as described previously [9]. Genes encoding enzymes and transporters were cloned into the p426 (2 μ) and pGREG (CEN/ARS) plasmids, respectively (Table S2). All the genes were expressed under the control of the *TEF1* promoter from *S. cerevisiae*. Plasmids were introduced by transformation into *S. cerevisiae* EBY.VW4000 following the LiAC/SS carrier DNA/PEG protocol [25]. Cultures transformed with these plasmids were plated onto SC plates lacking uracil and leucine. Primers used to construct the expression plasmids are listed in Table S3.

#### Construction of *K. marxianus* mutants

The CRISPR-Cas9 system was used for the construction of *cel2* and *lac4 K. marxianus* mutants. Target sequence identification was carried out using the sgRNAcas9 [26]. Primers encoding the target sequences and specific overhangs (Table S3) were annealed and cloned into pUCC001, as described previously [27, 28]. In brief, the two complementary oligonucleotides encoding the target sequence were combined and phosphorylated using T4 polynucleotide kinase. The resulting DNA duplex was then cloned into pUCC001 via Golden Gate assembly. The BSA-R primer was used in combination with the target forward primer to check for correct assembly of the plasmids in *E. coli*. Plasmids containing the target sequences were purified and introduced into *K. marxianus* NBRC1777 by transformation. Transformants were checked by PCR using diagnosis primers and plasmid curing was performed by growing the strains in YPD without Hygromycin B for 16 hours. The strains were then plated in YPD and three colonies were streaked in YPD + Hygromycin B to confirm plasmid loss. The genotype of the mutants constructed is shown in Table S4.

#### Genome sequencing

The type strain of *K. lactis* var. *drosophilarum* (CBS2105) was purchased from the Westerdijk Institute (the Netherlands) and its genome was sequenced using Illumina and Pacific Biosciences technology. For Illumina sequencing, genomic DNA was harvested from stationary-phase cultures by homogenization with glass beads followed by phenol-chloroform extraction and ethanol precipitation, and concentrated with the Genomic DNA Clean & Concentrator-10 (Zymo Research, catalog D4010). Illumina sequencing was done by BGI Tech Solutions (Hong Kong) on a HiSeq2500 instrument generating 150 bp paired-end reads. Illumina data was assembled using SPAdes version v3.11.1 [29], giving 65x coverage of the genome. Genomic DNA for PacBio sequencing was prepared as in [30]. PacBio sequencing was done by the Earlham Institute (UK) using a PacBio Sequel instrument (1 SMRT cell), and assembled by them using HGAP4 [31], producing 224x coverage. BLASTN alignments to the Illumina contigs were used to detect and correct single-base indel errors in the PacBio scaffolds. The *K. lactis* var. *drosophilarum* CBS2105 genome sequence was annotated using YGAP [32] and submitted to the NCBI/ENA/DDBJ database (GenBank: GCA_007993695.1). The *K. marxianus* L03 genome was sequenced and assembled as described in [7] and was filtered using the CVL method [33] with 20x kmer coverage and 5.5 kb length cut-offs before deposition in the NCBI/ENA/DDBJ database (GenBank: GCA_008000265.1).

#### Bioinformatic analysis

The phylogenetic tree in Figure 1 was produced using a set of 1,515 single-copy orthologous amino acid sequences obtained from genome assemblies of 9 *Kluyveromyces* type strains plus *Lachancea thermotolerans* using the saccharomyceta_odb9 dataset with BUSCO v3.1.0 [34]. Orthologous sequences were obtained from *K. marxianus* NBRC1777 (GenBank assembly accession: GCA_001417835.1 [35]), *L. thermotholerans* CBS6340^T^ (GCF_000142805.1), *K. aestuarii* CBS4438^T^ (GCA_003707555.1), *K. nonfermentans* CBS8778^T^ (GCA_003670155.1), *K. wickerhamii* CBS2745^T^ (GCA_000179415.1), *K. dobzhanskii* CBS2104^T^ (GCA_000820885.1), *K. lactis* var. *lactis* CBS2359^T^ (GCF_000002515.2), *K. lactis* var. *drosophilarum* CBS2105^T^ (GCA_007993695.1), *K. marxianus* L03 (GCA_008000265.1), and *K. marxianus* UFS-Y2791 (GCA_001692465.1). Each group of 10 orthologous sequences were aligned using CLUSTALΩ (v1.2.4 [36];) removing gaps with trimAl v1.2rev59 in -nogaps mode [37]. Trimmed multiple sequence alignments were then concatenated and used to calculate a phylogenetic tree with RAxML-NG v0.8.1 [38] using 10 random and 10 parsimony-based starting trees, to pick the best-scoring topology to then do 100 bootstrap replicates with the PROTGTR substitution model.

Global nucleotide sequence identity between the *K. lactis* var. *lactis* CBS2359 and *K. lactis* var. *drosophilarum* CBS2105 genomes was calculated using the nucmer program of the MUMmer package (version 3.23) [39], filtered to retain only alignments longer than 10 kb (option delta-filter –g –l 10000). The divergence time of the introgressed region was calculated assuming a mutation rate of 3e-10 per site per generation and 10-100 yeast generations per year, as in [19].

### Data and Code Availability

The accession numbers for the *K. lactis* var. *drosophilarum* CBS2105 and *K. marxianus* L03 genome assemblies reported in this paper are GenBank: GCA_007993695.1 and GenBank: GCA_008000265.1, respectively.

## Supplemental Information

Document S1. Figures S1–S4 and Tables S1–S4

Document S2. Article plus Supplemental Information
