# Supplementary material for: Origin of Lactose Fermentation in Kluyveromyces lactis by Interspecies Transfer of a Neo-functionalized Gene Cluster during Domestication
Source: Curr Biol. 2019 Dec 16;29(24):4284–4290.e2. doi: 10.1016/j.cub.2019.10.044 (PMC6926475; doi:10.1016/j.cub.2019.10.044)
Supplement: Document S1. Figures S1–S4 and Tables S1–S4 [file mmc1.pdf]

Current Biology, Volume *29*

Supplemental Information

Origin of Lactose Fermentation in *Kluyveromyces lactis* by Interspecies Transfer of a Neo-functionalized Gene Cluster during Domestication

Javier A. Varela, Martina Puricelli, Raúl A. Ortiz-Merino, Romina Giacomobono, Stephanie Braun-Galleani, Kenneth H. Wolfe, and John P. Morrissey

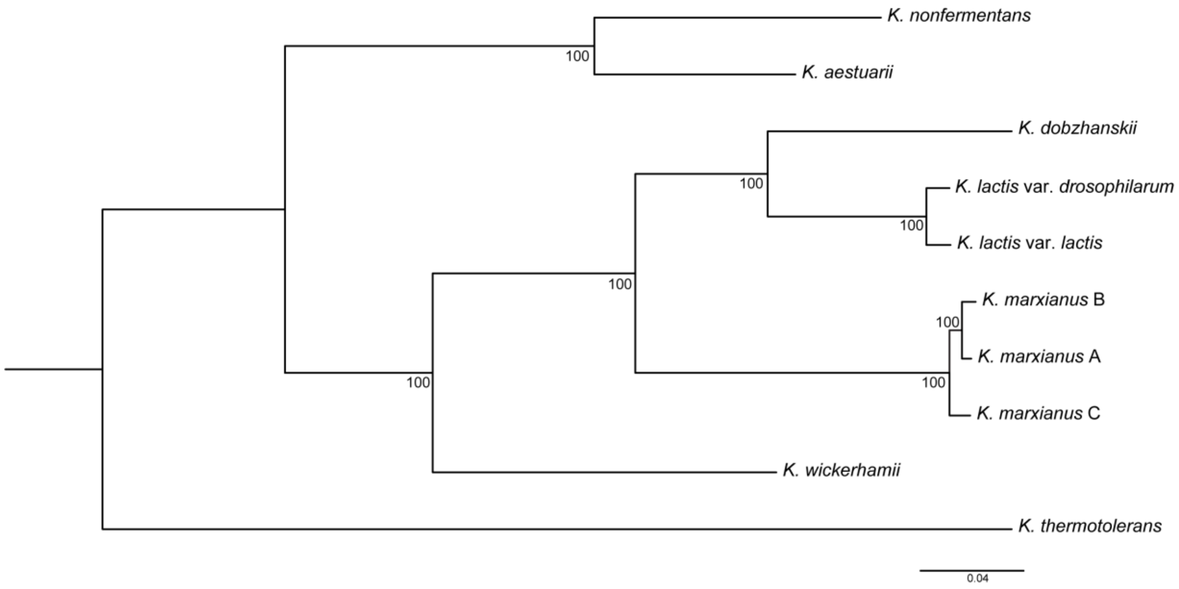

Figure S1. Phylogeny of *Kluyveromyces* species. Related to Figure 1. The tree was constructed using 1,515 single-copy orthologous amino acid sequences from *Kluyveromyces* genome assemblies (see STAR Methods). The values displayed on the branches correspond to bootstrapping values for the tree. The tree was computed with 100 bootstrap replicates.

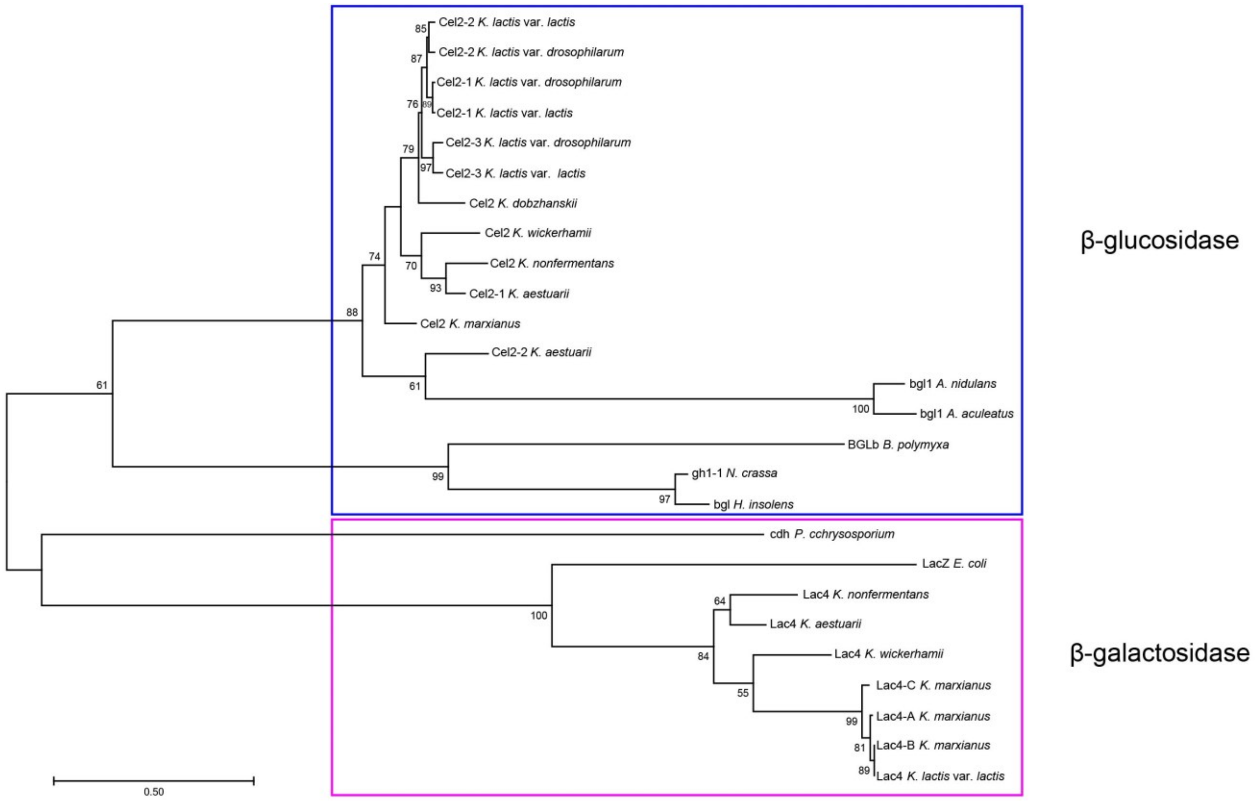

Figure S2. Phylogenetic tree of β-glucosidases and β-galactosidases. Related to Figure 3. Cel2 proteins from *Kluyveromyces* species cluster with β-glucosidases from other fungi, whereas Lac4 proteins form a clade with the *E. coli* LacZ β-galactosidase. The maximum likelihood tree was computed using MEGA 7 [S1] with 500 bootstrap replicates. Branches with >50% bootstrap support are labeled.

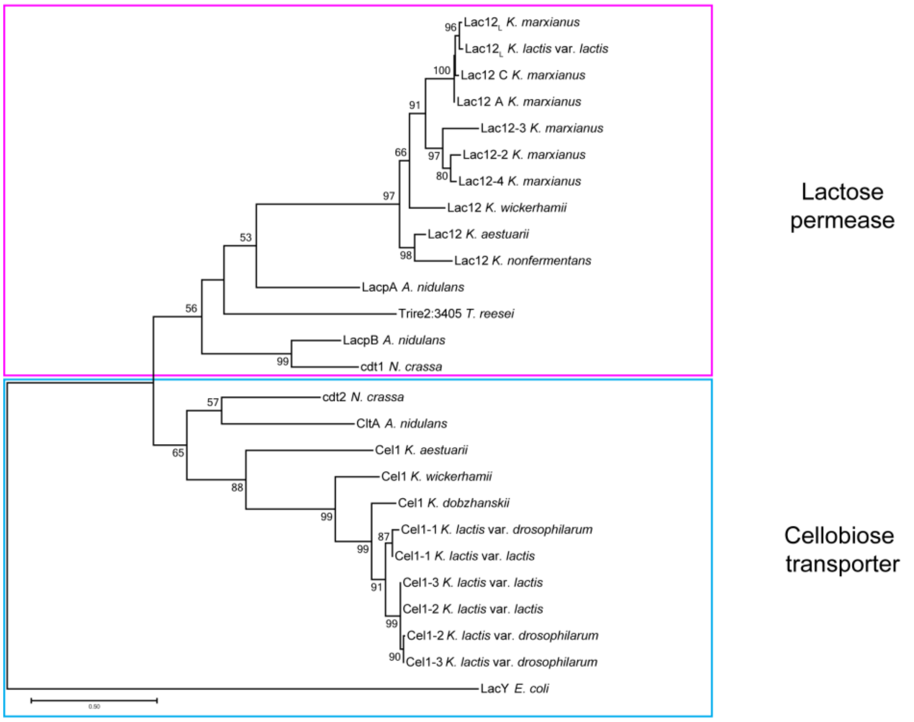

Figure S3. Phylogenetic tree of cellobiose and lactose transporters. Related to Figure 3. The tree was drawn following a multiple sequence alignment of Lac12 and Cel1 proteins and their homologs in other species. Cel1 clusters with known fungal cellobiose transport genes, whereas Lac12 is in a clade with known fungal lactose permeases. The *E. coli* LacY sequence was used as outgroup. The maximum likelihood tree was computed using MEGA 7 [S1] with 500 bootstrap replicates. Branches with >50% bootstrap support are labeled.

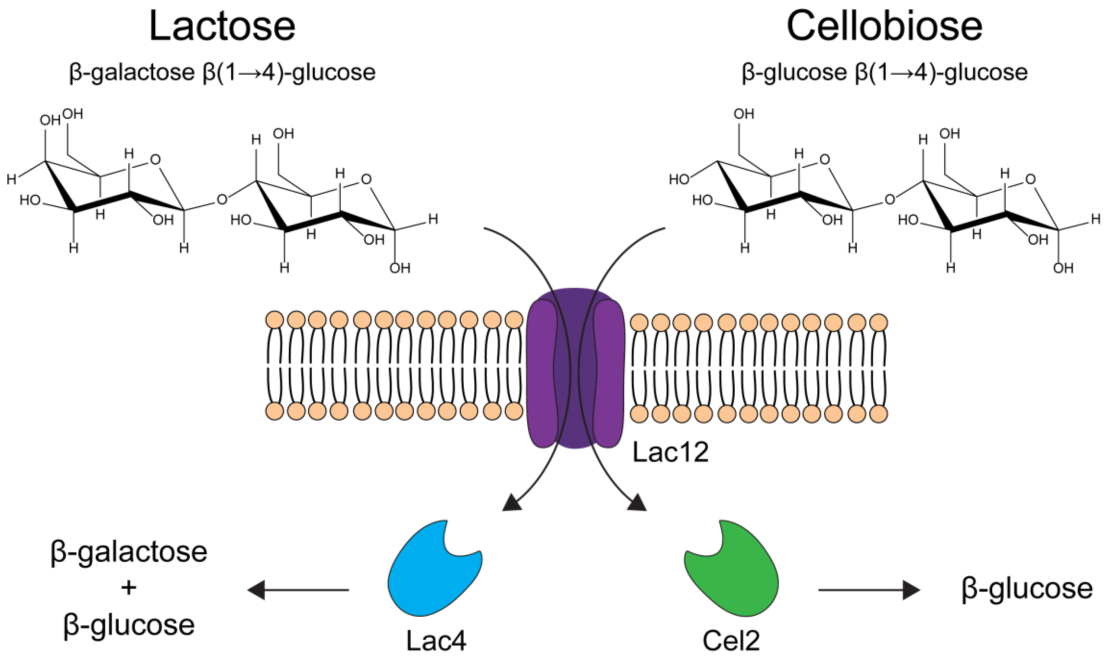

Figure S4. Scheme showing the bifunctional behavior of Lac12 from *K. marxianus.* Related to Figure 4. The absence of Cel1, a dedicated cellobiose transporter in *K. marxianus,* is compensated by Lac12 that is able to transport either lactose or cellobiose. After being transported into the cell via Lac12, lactose and cellobiose are hydrolyzed into monomers by Lac4 (lactase) and Cel2 (cellobiase), respectively.

Species	Source
K. aestuarii CBS4438^{T}	Westerdijk Fungal Biodiversity Institute
K. dobzhanskii CBS2104^{T}	Westerdijk Fungal Biodiversity Institute
K. lactis var. *lactis* CBS2359^{T}	Westerdijk Fungal Biodiversity Institute
K. lactis var. *drosophilarum* CBS2105^{T}	Westerdijk Fungal Biodiversity Institute
K. marxianus CBS397	Westerdijk Fungal Biodiversity Institute
K. marxianus NBRC1777	NITE Biological Research Centre
K. marxianus NBRC1777 *cel2*	This study
K. marxianus NBRC1777 *lac4*	This study
K. marxianus NBRC1777 *cel2 lac4*	This study
K. marxianus NBRC1777 *lac12 lac12-2 lac12-4*	[S2]
K. wickerhamii CBS2745^{T}	Westerdijk Fungal Biodiversity Institute
S. cerevisiae EBY.VW4000	Goethe University Frankfurt, Germany [S3]

Table S1. Yeast strains used in this study. Related to Figure 1 - 4. ^{T} indicates when the strain used is type strain for the species [S4].

Plasmid	Source
pGREG-505-TEF1	[S5]
pGREG-*KmLAC12* CBS397	[S5]
pGREG-*KmLAC12-2* CBS397	[S5]
pGREG-*KmLAC12-3* CBS397	[S5]
pGREG-*KmLAC12-4* CBS397	[S5]
pGREG-*KaLAC12* CBS4438	This study
pGREG-*KwLAC12* CBS2745	This study
pGREG-*KdCEL1* CBS2104	This study
p426	Addgene #43803
p426-TEF1-*KdCEL2* CBS2104	This study
p426-TEF1-*KwLAC4* CBS2745	This study
pUCC001	[S2]
pUCC001-*KmLAC4*	[S6]
pUCC001-*KmCEL2*	This study
pUCC001-*KmLAC12*	[S2]
pUCC001-*KmLAC12-2*	[S2]
pUCC001-*KmLAC12-4*	[S2]

Table S2. Plasmids used in this study. Related to Figure 4.

Heterologous expression

Name	Sequence
p426-F	cttagattagattgctatgctttc
p426-R	tcatgtaattagttatgtcacgc
pGREG505-F	cctcgagtcatgtaattagttatgtcacgc
pGREG505-R	cgacggtatcgataagcttgatatcgaa
KaLAC12-F	gaattcgatatcaagcttatcgataccgtcgatgctagaacagagttcaagttct
KaLAC12-R	gcgtgacataactaattacatgactcgaggtcgacttagaccttattctcgatttgttggt
KwLAC12-F	gaattcgatatcaagcttatcgataccgtcgatgctccaagtccatcaaatca
KwLAC12-R	gcgtgacataactaattacatgactcgaggtcgactcatgcggattcggattcca
KdCEL1-F	gaattcgatatcaagcttatcgataccgtcgatgtcaaaggattcaatcttagatagtg
KdCEL1-R	gcgtgacataactaattacatgactcgaggtcgactcaattaatcgcttccttccttt
KdCEL2-F	gcatagcaatctaatctaagatgtcaaatttcgatgtcgaacaa
KdCEL2-R	tgacataactaattacatgactacaacccggtccagtaag
KwLAC4-F	gcatagcaatctaatctaagatgtcaacccaattgattccttc
KwLAC4-R	tgacataactaattacatgactactcaaaaccaatgttgaaggt

Mutant construction

Name	Sequence
KmCEL2-gRNA-F	cgtcacggtatgttaatgtccgac
KmCEL2-gRNA-R	aaacgtcggacattaacataccgt
KmLAC4-gRNA-F	cgtcgacatctcttaggacaagtt
KmLAC4-gRNA-R	aaacaacttgtcctaagagatgtc
Diag-*KmCEL2*-F	ttgttaaaggtatgcagggc
Diag-*KmCEL2*-R	ggatgctgaaccaccac
Diag-*KmLAC4*-F	cttaattagcttgtacatggct
Diag-*KmLAC4*-R	tggatttaattggatctgatgg
BSA-R	tacacgcgtttgtacagaaaaaaagaaaaatttga

Table S3. Primer sequences used in this study. Related to Figure 4.

Strain	Mutation coordinates	Source
NBRC1777 *lac12 lac12-2 lac12-4*	*KmLAC12:*CHR III 16,869..-T<br>*LAC12-2:* CHR III 1,557,231..-T<br>*LAC12-4:* CHR VIII 12,834..-GT	[S2]
NBRC1777 *cel2*	CHR III Δ20,048 – 20,054	This study
NBRC1777 *lac4*	CHR III 11,255..-A	This study
NBRC1777 *cel2 lac4*	*CEL2:* CHR III Δ20,048 – 20,054<br>*LAC4:* CHR III 11,255..-A	This study

Table S4. Genotype of the *K. marxianus* mutants constructed in this study. Related to Figure 4. Coordinates of the mutations in the NBRC1777 genome are shown. The nucleotides deleted are represented by a (-) symbol or Δ in cases where several nucleotides were deleted.

Supplemental References

S1. Kumar, S., Stecher, G., and Tamura, K. (2016). MEGA7: Molecular Evolutionary Genetics Analysis Version 7.0 for Bigger Datasets. Mol. Biol. Evol. *33*, 1870–1874. Available at: http://www.ncbi.nlm.nih.gov/pubmed/27004904

S2. Varela, J.A., Puricelli, M., Montini, N., and Morrissey, J.P. (2019). Expansion and Diversification of MFS Transporters in *Kluyveromyces marxianus*. Front. Microbiol. *9*, 3330. Available at: http://www.ncbi.nlm.nih.gov/pubmed/30687296

S3. Wieczorke, R., Krampe, S., Weierstall, T., Freidel, K., Hollenberg, C.P., and Boles, E. (1999). Concurrent knock-out of at least 20 transporter genes is required to block uptake of hexoses in *Saccharomyces cerevisiae*. FEBS Lett. *464*, 123–8. Available at: http://www.ncbi.nlm.nih.gov/pubmed/10618490

S4. Lachance (2011). *Kluyveromyces van der Walt.* In: Kurtzman C.P., Fell J.W., Boekhout T. (eds) The yeasts : A taxonomic study, Elsevier, Amsterdam, pp. 471-481

S5. Varela, J.A., Montini, N., Scully, D., Van der Ploeg, R., Oreb, M., Boles, E., Hirota, J., Akada, R., Hoshida, H., and Morrissey, J.P. (2017). Polymorphisms in the *LAC12* gene explain lactose utilisation variability in *Kluyveromyces marxianus* strains. FEMS Yeast Res. *17*. Available at: http://www.ncbi.nlm.nih.gov/pubmed/28444380

S6. Rajkumar, A.S., Varela, J.A., Juergens, H., Daran, J.-M.G., and Morrissey, J.P. (2019). Biological Parts for *Kluyveromyces marxianus* Synthetic Biology. Front. Bioeng. Biotechnol.
